# Comparative evaluation of tractography-based direct targeting and atlas-based indirect targeting of the ventral intermediate (Vim) nucleus in MRgFUS thalamotomy

**DOI:** 10.1038/s41598-021-93058-2

**Published:** 2021-06-29

**Authors:** Federico Bruno, Alessia Catalucci, Marco Varrassi, Francesco Arrigoni, Patrizia Sucapane, Davide Cerone, Francesca Pistoia, Silvia Torlone, Emanuele Tommasino, Luca De Santis, Antonio Barile, Alessandro Ricci, Carmine Marini, Alessandra Splendiani, Carlo Masciocchi

**Affiliations:** 1 Department of Biotechnological and Applied Clinical Sciences, University of L’Aquila, Via Vetoio 1, 67100 L’Aquila, Italy; 2 Italian Society of Medical and Interventional Radiology, SIRM Foundation, Milan, Italy; 3 Neuroradiology and Interventional Radiology, San Salvatore Hospital, L’Aquila, Italy; 4 Neurology, San Salvatore Hospital, L’Aquila, Italy; 5 Neurosurgery, San Salvatore Hospital, L’Aquila, Italy

**Keywords:** Neurology, Parkinson's disease

## Abstract

To analyze and compare direct and indirect targeting of the Vim for MRgFUS thalamotomy. We retrospectively evaluated 21 patients who underwent unilateral MRgFUS Vim ablation and required targeting repositioning during the procedures. For each patient, in the three spatial coordinates, we recorded: (i) indirect coordinates; (ii) the coordinates where we clinically observed tremor reduction during the verification stage sonications; (iii) direct coordinates, measured on the dentatorubrothalamic tract (DRTT) at the after postprocessing of DTI data. The agreement between direct and indirect coordinates compared to clinically effective coordinates was evaluated through the Bland–Altman test and intraclass correlation coefficient. The median absolute percentage error was also calculated. Compared to indirect targeting, direct targeting showed inferior error values on the RL and AP coordinates (0.019 vs. 0.079 and 0.207 vs. 0.221, respectively) and higher error values on the SI coordinates (0.263 vs. 0.021). The agreement between measurements was higher for tractography along the AP and SI planes and lower along the RL planes. Indirect atlas-based targeting represents a valid approach for MRgFUS thalamotomy. The direct tractography approach is a valuable aid in assessing the possible deviation of the error in cases where no immediate clinical response is achieved.

## Introduction

Surgical treatment for tremor, in patients with essential tremor (ET) and Parkinson's disease (PD), can be considered in cases resistant to drug therapy^1, 2^. Currently available options include Deep Brain Stimulation (DBS), radiofrequency (RF) thalamotomy, and radiation therapy (namely Gamma-Knife thalamotomy). In recent years, Magnetic Resonance guided Focused Ultrasound Surgery (MRgFUS) has also been successfully applied to the minimally-invasive treatment of medically refractory tremors^3, 4^, and several studies confirmed its safety and efficacy.

All functional neurosurgical procedures are directed to specific anatomical areas involved in the neurofunctional circuits of motion control; the intermediate ventral nucleus (Vim) of the thalamus is one of the main targets of choice for patients with ET and patients with tremorgenic PD^5, 6^. However, the anatomical detection of the Vim is complicated and limited even with high-resolution MRI sequences on high-field scanners, as the thalamus lacks sufficient intrinsic contrast^7^. Histologically, the Vim measures about 4 mm in the anteroposterior dimension, 4 mm mediolaterally, and 6 mm dorsoventrally, representing approximately 0.5–2.0% of the total thalamic volume^8–10^. To date, the routinely used method for intraprocedural detection of Vim in practice is indirect targeting, based on histological atlases. However, this method, being carried out based on anatomical landmarks (namely the anterior and posterior commissure, AC-PC), is not patient-specific and is thus relatively insensitive to interindividual anatomical variations^11–14^. Moreover, wide variability and differences exist in the indirect targeting coordinates reported by the various centers: commonly used coordinates are 13–15 mm lateral to the midline, 6 mm posterior to the mid-commissural point, or anterior to the posterior commissure, and 0–2 mm above the AC-PC line^15^.

Direct targeting is based on the target point anatomic visualization and thus tailored to the individual patient's anatomy. Regarding Vim targeting, there is a growing body of literature that recognizes MR diffusion tensor imaging (DTI) with tractography of the dentatorubrothalamic tract (DRTT) as one of the most valid methods for in vivo imaging visualization^16, 17^. So far, however, few studies have investigated the actual error of the two techniques by directly comparing tractography-based targeting and atlas-based Vim targeting.

Therefore, the purpose of our study was to analyze and compare direct and indirect targeting of the Vim for MRgFUS thalamotomy in patients with ET and PD, using clinically effective intraprocedural coordinates as a "gold standard" control.

## Results

The mean values of the indirect coordinates were 7.47 ± 0.46 mm (6.5–8.4 mm) on the AP plane, 14.15 ± 0.45 mm (13-15 mm) mm on the RL plane, and 1.15 ± 0.46 mm (0–2 mm) on the SI plane. The mean number of target shifts from the initial indirect coordinates to obtain clinical benefit was 3.2 ± 1.94 (1–7). The mean values of the target repositioning along the x, y, and z coordinates were respectively 0.66 mm along the RL plane, 0.48 mm along the AP plane, and 0.32 mm along the SI plane. The mean values of the target repositioning direction were 0.19 mm medially and 0.11 mm laterally along the RL plane, and 0.26 mm anterior and 0.35 mm posterior along the AP plane; 0.18 mm cranially and 0.16 mm caudally on the SI plane.

The mean values of the clinically effective coordinates were 7.26 ± 0.8 mm (6–8.8 mm) on the AP plane, 14.06 ± 0.63 mm (13–15.4 mm) on the RL plane, and 1.23 ± 0.6 mm (0–2.5 mm) on the SI plane.

The mean values of the direct coordinates were 7.43 ± 0.78 mm (5.9–8.8 mm) on the AP plane, 14.08 ± 1.09 mm (11.3–16.5 mm) on the RL plane, and 1.41 ± 0.62 mm (0.39–2.9 mm) on the SI plane.

**Figure 1:**
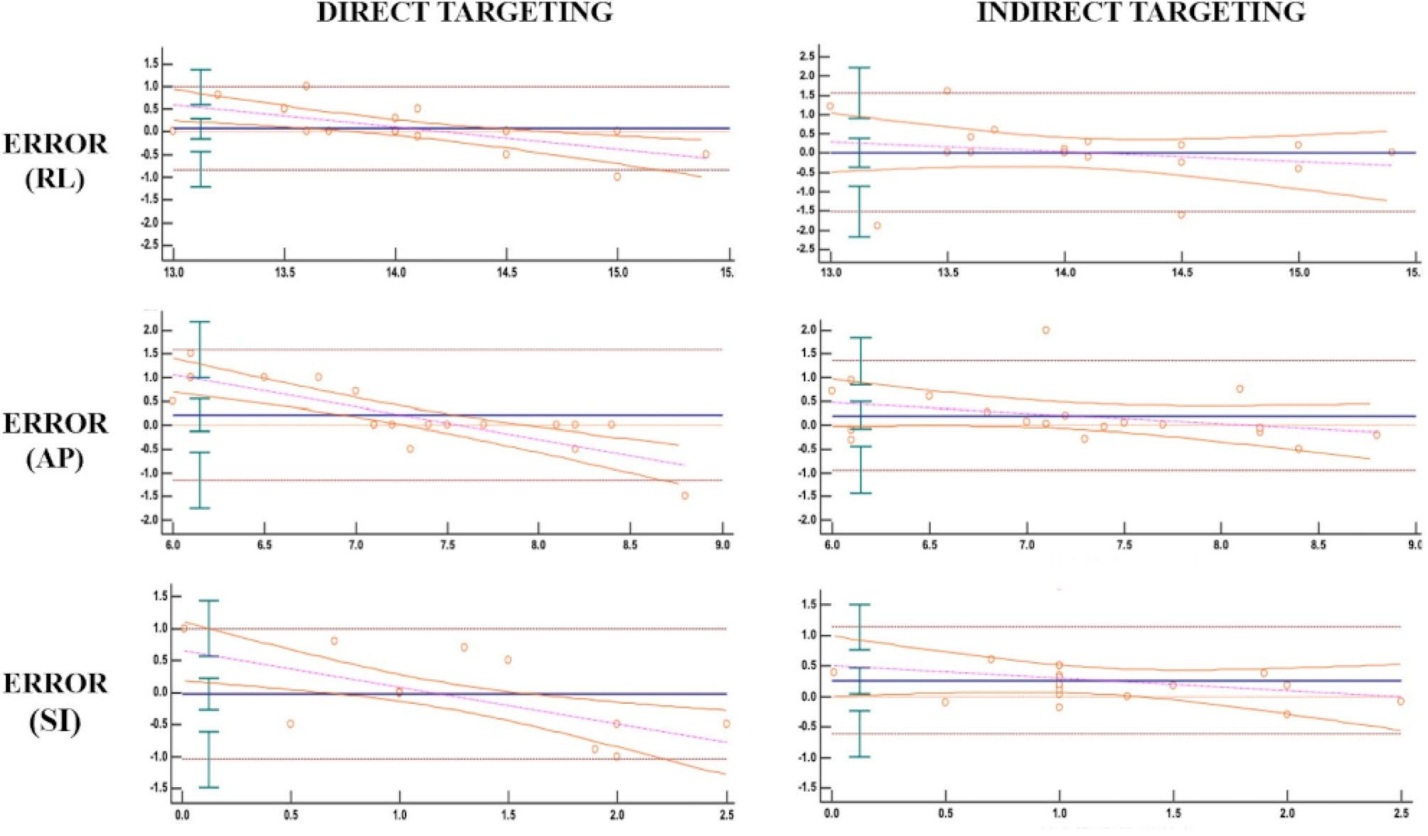
Bland Altman plots for direct and indirect targeting.

The Bland–Altman analysis showed that the mean error of the difference between the direct and indirect coordinates compared to the clinically effective coordinates was respectively 0.207 and 0.221 along the AP direction, 0.019 and 0.079 along the RL, and 0.263 and 0.021 along the SI direction (Table 1, Fig. 1)).

**Table 1:** Detailed results of Bland-Altaman analysis with mean error of the differences between the direct and indirect coordinates compared to the clinically effective coordinates.

	Indirect targeting	Direct targeting
Error (RL)	0.08 ± 0.46 (95% CI − 0.14–0.30)	0.02 ± 0.78 (95% CI − 0.36–0.40)
Error (AP)	0.22 ± 0.70 (95% CI − 0.11–0.56)	0.21 ± 0.58 (95% CI 0.86–1.83)
Error (SI)	0.02 ± 0.52 (95% CI − 0.27–0.23)	0.26 ± 0.45 (95% CI − 0.05–0.48)

The ICC analysis (Table 2) showed:

**Table 2:** Detailed results of ICC analysis.

Indirect targeting	ICC (single measures)	ICC (mean)	Indirect targeting	ICC (single measures)	ICC (mean)
SI	0.51 (95% CI 0.09–0.79)	0.68 (95% CI 0.18–0.88)	SI	0.68 (95% CI 0.29–0.85)	0.81 (95% CI 0.45–0.92)
RL	0.64 (95% CI 0.31–0.85)	0.78 (95% CI 0.47–0.92)	RL	0.51 (95% CI 0.07–0.76)	0.68 (95% CI 0.12–0.87)
AP	0.46 (95% CI 0.06–0.75)	0.63 (95% CI 0.11–0.86)	AP	0.76 (95% CI 0.48–0.88)	0.87 (85% CI 0.65–0.93)

The agreement between the indirect and clinically effective coordinates (mean values) was:

- ICC = 0.632 along the AP plane (95% CI 0.12–0.86)
- ICC = 0.785 along the RL plane (95% CI 0.47–0.92)
- ICC = 0.681 along the SI plane (95% CI 0.18–0.88)

The agreement between the direct and clinically effective coordinates (mean values) was:

- ICC = 0.871 along the AP plane (95% CI 0.65–0.93)
- ICC = 0.680 along the RL plane (95% CI 0.12–0.87)
- ICC = 0.807 along the SI plane (95% CI 0.45–0.93)

## Discussion and conclusions

Indirect targeting for preoperative planning in functional neurosurgical procedures has extensively been debated in the scientific literature. Most of the published experience on Vim identification based on stereotaxic neurosurgical atlases relates to surgical thalamotomy procedures (i.e. radiofrequency, Gamma-knife, and DBS)^12, 16, 18–21^, but most of the same approaches have also been translated to MR guided focused ultrasound thalamotomy procedure. Despite the numerous attempts to obtain univocal and precise coordinates for Vim targeting, many authors continued to find differences between the target of indirect coordinates and the clinical target, especially in the RL orientation, with a mean error of about 2 mm^14, 22^. Other authors have observed errors up to 5 mm using indirect targeting, associated with even serious adverse events^12^. For these reasons, authors have tried to approach personalized stereotactic coordinates based on the patient's anatomy, laying the foundation for what today is called direct targeting. In this scenario, advanced MR sequences, such as quantitative susceptibility mapping (QSM), fast gray matter acquisition T1 inversion recovery (FGATIR) and WAIR (white matter attenuated inversion recovery), that improve the ability to image the Vim region, are emerging^23^. However, they have not yet been shown to have reliability and accuracy to serve as the primary method of Vim targeting^24, 25^. Currently, the most promising imaging approach to directly identify the Vim region for clinical purposes is MR diffusion tensor imaging and tractography^26–30^. In 2011, Coenen et al. published the first successful results regarding the surgical introduction of stimulating intracranial electrodes in the Vim region through tractography for the treatment of ET^31^. However, the accuracy of DRTT deterministic tractography reconstruction was assessed to be liable to an error range between 1–3 mm^32^. The possible reasons were probably related to the echo-planar MRI sequences used for fiber reconstruction, as they were susceptible to distortions caused by eddy currents with large diffusion gradients^33^. In 2016, Sammartino et al. proposed defining specific tractography landmarks to identify the thalamic ROI for the DRT tract reconstruction using a deterministic algorithm. These landmarks included selecting a ROI lateral to the pyramidal tract and posterior to the medial lemniscus, with a 3 mm distance from these structures. The DRT tractography reconstructions obtained in this way have found confirmations by neurophysiological and clinical evaluation after DBS^17, 34^.

The validity of the DRTT tractography in Vim targeting for tremor treatment was also confirmed using MRgFUS, demonstrating the postoperative evidence of DRTT interruption in patients with clinical response^35, 36^. Several tractography targeting systems have been described to reduce the Vim localization error as much as possible. Krishna et al. have determined the possibility of detecting Vim through the reconstruction of two fiber tracts: the pyramidal and somatosensorial tracts using the inferential method^29^. Chazen et al. have used tractography to detect Vim in preoperative planning for MRgFUS thalamotomy mapping the dentatorubrothalamic tract (DRTT), the corticospinal tract (CST), and medial lemniscus (ML)^36^. This algorithm, called "three tracts tractography"^37^, has also been used by Lehman et al.^26^ for Vim targeting in the planning before MRgFUS and DBS.

However, very little was found in the literature on the direct comparison between atlas-based and DTI targeting approach in MRgFUS Vim thalamotomy. In a retrospective report of 4 ET patients, Miller et al.^38^ compared the location of the 24-h thalamic lesion with the center of the stereotactic coordinates, and the overlap between lesion size and the DRTT, founding a divergence > 1 mm between indirect and direct coordinates in all cases in both the mediolateral and the anteroposterior plane. Another study by Krishna et al.^29^ evaluated the short-term (3 months) procedural outcome in 9 ET patients submitted to MRgFUS with prospective tractography based Vim targeting. Comparing DRTT tractography and indirect coordinates, they found a significant difference of about 1 mm in the anteroposterior direction. The present study differs from these previous reports in several aspects. In addition to evaluating a larger number of cases, we compared direct coordinates, obtained by probabilistic single tract tractography mapping of the DRTT, and indirect atlas-based coordinates, using a "gold standard" targeting (i.e., the coordinates were we actually obtained clinically effective tremor reduction intraprocedurally). Moreover, the comparison using the amount of DRT tract included in the ablation lesion in our opinion could be little indicative of the matching with the indirect coordinates, since the diameter of the lesion (mean reported volume in literature, about 300–400mm^3^^39–43^) is much higher than the spatial resolution of the intra-procedural target shifts using MR guidance (0.1 mm). Moreover, the final lesion configuration and extent depend on many individual patient factors, the most relevant being, among others, the skull density ratio (SDR) values and the number of active transducers^29, 38^.

Although many authors have indeed used d-DRTT (decussating-DRTT) for procedures of Vim targeting, and despite the only recent identification of nd-DRTT (nondecussating-DRTT), in the present study we used the ipsilateral nd-DRTT as the main WM tract for Vim targeting. Some recent studies demonstrates that despite d-DRTT composes approximately 80% of DRTT fibers, the nd-DRTT reaches predominantly (according to d-nd ratio) 4 of the total 8 thalamic nuclei reached by DRTT (with not negligible amount of intermingling between nd-DRTT and d-DRTT reaching thalamic GM, which yet gives reason to the impossibility of defining exact segregation of all fibers, especially around the Vim). Among these four nuclei, the ones which define better VLP (which corresponds to Hassler Vim) are VPL (placed posteriorly and inferiorly, which is reached predominantly by nd-DRTT fibers d/nd ratio 0.8) and VLA (d-nd ratio 1.2), judging by the work of Kalen J. Petersen et al.^44^. VLP would be the ideal correspondent of Vim, however exact matching with nd-DRTT and/or d-DRTT termination is not clear enough yet. Evidence of this is reported in some studies identifying nd-DRTT fibers reaching VLP (and also VLA, which is notorious for its non-negligible degree of GM spatial interlocking with VLP), which would reasonably lead to the consideration of nd-DRTT being a good enough conductor to the target^44, 45^.

The hypothesis of nd-DRTT being the best conductor to Vim target might also find reasonable evidence in studies reporting an error occurrence of about 2.5 mm in procedures of MrgFUS thalamotomy using d-DRTT as the only conductor to Vim target^46^ and also from the work by Chazen et al. showing immediate postprocedure DTI failing to track DRTT ipsilateral to the lesion site with a preserved contralateral DRTT coincident with a substantial resolution of contralateral tremor (validating the relationship between the tremor reduction and nd-DRTT interruption at least)^36^.

On the other hand, other studies report the Vim as being located at the exact point of anterior–posterior fading between nd-DRTT fibers and d-DRTT ones at AC-PC level^44^. This would give reason to the consideration that both the DRTT components would be eligible for targeting the Vim, respectively the most posterior part of d-DRTT and the most anterior of the nd-DRTT (has to be also considered that nd-DRTT and d-DRTT have diameters which depend on the patient, but rarely inferior to 2.0 mm, without considering the highest spatial resolution of tractography which is around 1–2 mm in the best cases, exception made for ultra-high-field strengths which can reach 0.33 mm resolution, but are not eligible for in vivo studies)^47^.

Another result included in our analysis, not evaluated in previous works, is the measurement of coordinates in the craniocaudal (SI) plane. We believe that accuracy of target assessment on the craniocaudal direction is crucial since some of the procedural side effects (namely, ataxia) are due to the inadvertent inferior extension of the edema/ablation^2^. The Bland–Altman analysis showed that the error was slightly inferior for direct targeting in our population compared to indirect targeting on the RL and AP coordinates; instead, it was inferior on the SI coordinates for indirect targeting. The ICC tests demonstrated an excellent agreement between the tractography values and the clinical response coordinates along the AP and SI planes, and a moderate agreement along the RL planes. Furthermore, the agreement between indirect and "gold standard" coordinates was excellent along the RL plane and moderate along the AP and SI planes. On the SI plane, the indirect targeting error was smaller; we explained this finding considering the elongated anatomic Vim configuration on the caudal-cranial direction and that we might similarly have a clinical response (tremor reduction) sonicating either cranial or caudal portions of the Vim. Conversely, the direct targeting ICC appeared excellent on the SI plane, while it was smaller on the RL plane. This happened because rare targeting repositioning from the indirect coordinates is usually performed during treatments on the RL plane, due to the internal capsule proximity. In our experience, in cases with no clinical tremor reduction using initial indirect coordinates, we usually perform the first targeting movement attempt posteriorly, and this is probably why the indirect targeting of ICC on the AP line was lower than the direct one. Our intraprocedural strategy also includes the evaluation of thermometric maps to identify non-sonicated areas, and the retrospective evaluation of the mismatch between coordinate measures.

In our comparative evaluation, tractography-based direct coordinates to target the Vim during MRgFUS thalamotomy were slightly precise than commonly adopted indirect coordinates, relative to clinically effective coordinates, while resulting less accurate along the SI plane. It should also be considered that indirect targeting, although it may sometimes be more precise and accurate, does not provide any indication of the directionality of movement. This is a critical limitation, especially in patients with low SDR values, where it is essential to optimize the number of sonications^48, 49^.

The optimization of the number of sonications could also increase the precision of the ablation lesion size and potentially prevent complications and tremor relapses.

Another aspect, which will be supported by future studies, is that we did not apply tractography during treatment, as we performed a retrospective assessment. However, such a preliminary assessment would be beneficial in the course of treatment to suggest target movements, even when starting with an indirect approach. Nevertheless, technological advances will certainly allow, in the very near future, to integrate coils capable of acquiring DTI sequences of sufficient quality within the helmet, and, therefore, perform direct tractography-based targeting on intraprocedural acquisitions.

Our results are indeed limited by being deducted from a relatively small number of patients (representing 21.4% of all treated patients) where intra-procedural shifts of the target were required. The correlation of the targeting methods with the long-term clinical outcome was also beyond the scope of the present research, but we included in our series only patients with stable mid-term (at least six months) tremor reduction. The choice to select this latter clinical follow-up parameter is also due to the well-known tendency of patients with PD to present tremor relapses more frequently than patients with essential tremor.

In conclusion, indirect atlas-based targeting represents a valid approach for MRgFUS thalamotomy, allowing an immediate identification of the correct target in most cases. The direct tractographic approach is currently a valuable aid in assessing the possible deviation of the error in cases where no immediate clinical response is achieved. Therefore the full use and referral to both methods may be the best approach for Vim targeting at present.

## Methods

We retrospectively evaluated 98 patients with disabling and refractory tremors who underwent unilateral MRgFUS Vim ablation in the period between February 2018—October 2020 at our Institution. From procedural reports, we retrieved patients who required targeting repositioning during the procedures due to absent or insufficient clinical responses (i.e., tremor reduction). Other inclusion criteria were: (i) successful treatment (i.e., tremor improvement at the end of the procedure—defined as > 50% CRST score reduction respect to baseline—with stable effects at six months and absence of thalamotomy-related complications); ii) availability of pre-procedural MRI imaging, including DTI and 3D T1 sequences; iii) availability of complete procedural reports (e.g., description of intraprocedural sonication parameters, target coordinates, clinical events). The final study sample consisted of 21 patients (13 ET, eight PD, 15 males, seven females, mean age 64.2 ± 8.9 years). Mean pre- and post-treatment CRST score was 30 (range 9–48, 95% CI 26–34) and 11 (range 0–22, 95%CI 9.1–14), respectively. The left Vim was treated in 15 patients, right Vim in seven patients.

**Figure 2:**
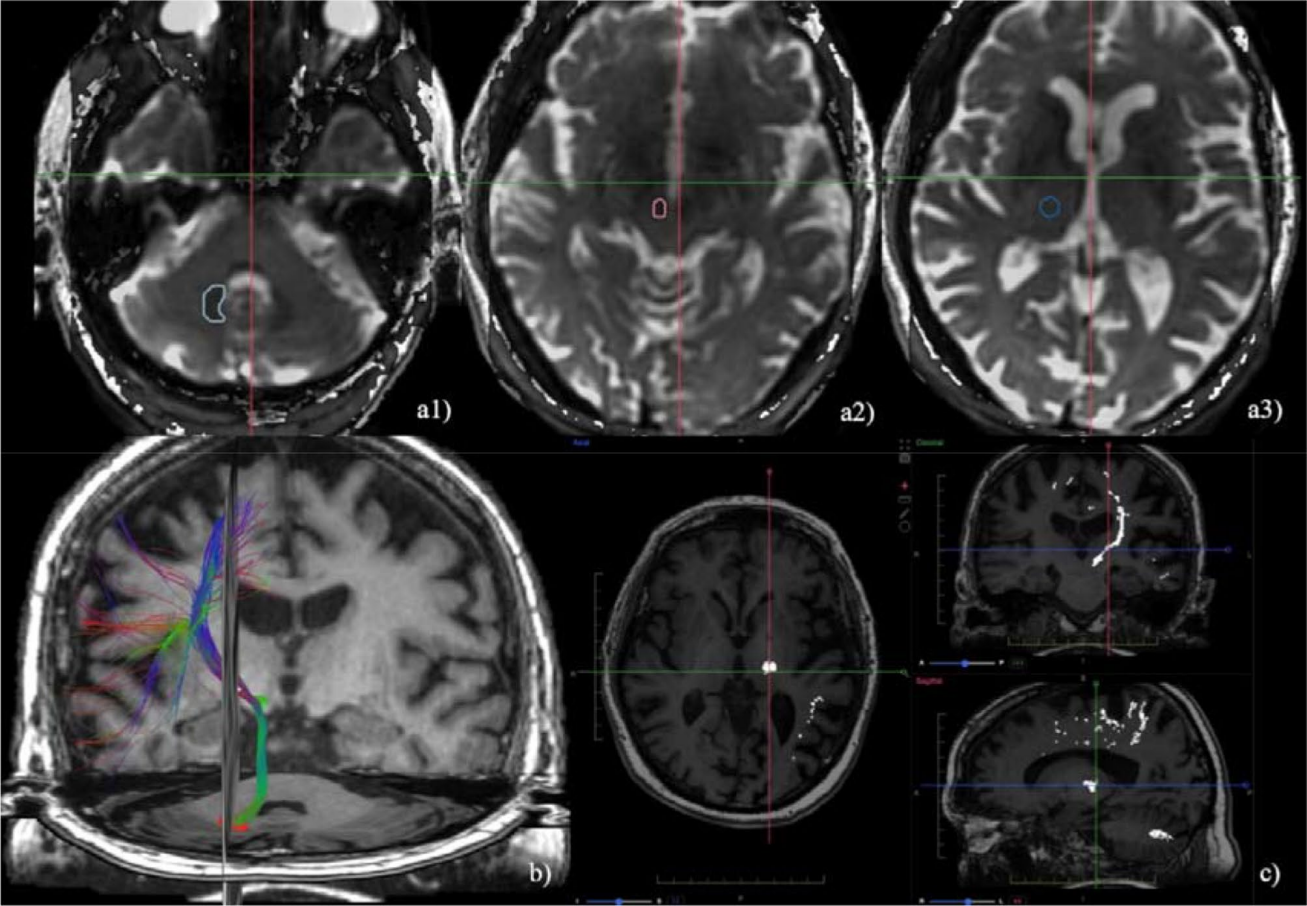
DTI tractography of the dentatorubrothalamic tract (DRTT). Manual definition of three regions of interest (ROIs) on axial images: the cerebellar dentate nucleus ipsilateral to the target (**a1**), the ipsilateral red nucleus (**a2**), and the supposed location of the ipsilateral Vim at the level of the thalamus on the AC-PC plane (**a3**). Fiber tractography 3D (**b**) and multiplanar 2D (**c**) visualization of the DRTT.

For each patient, we recorded:

1. Indirect coordinates (as recorded in the procedural reports). At our Institution, we set indirect targeting as follows: (i) on the anteroposterior (AP) direction, the mean distance between 25–30% of the intercommisural distance, anterior to PC; (ii) on the mediolateral (RL) direction, 14 mm laterally from the midline (or 11 mm laterally from the wall of the third ventricle); in case of a mismatch, we set the target halfway between the two measurements; (iii) on the superoinferior (SI) direction, 1–2 mm above the AC-PC line; during our initial experience, we set the target 1 mm cranially; however, due to the occasional caudal extension of the ablation and/or edema, we currently prefer to set the initial target 2 mm above the AC-PC line;

(2) The coordinates where we clinically observed tremor reduction during the verification stage sonications (as recorded in the procedural reports);

(3) Direct coordinates, measured on the dentatorubrothalamic tract (DRTT) after DTI data postprocessing, as described below. All MRI examinations were performed on a 3 T scanner (*Discovery 750w, GE Healthcare*) using a 32-channel head coil before the preoperative planning procedure. DTI sequences were acquired using the following parameters: 33 diffusion directions, TR 5700 ms, TE 98 ms, parallel imaging (acceleration factor two), 3 mm slice thickness, 39 slices, matrix 128 × 128, 230 mm FOV, b value 1000 s/mm2, acquisition time 4:01 min. A T1-weighted 3D IR FSPGR BRAVO sequence with multiplanar reconstructions was also acquired (parameters: FOV 24, slice thickness 1.6 mm, flip angle 20°, prep time 450, TE 3.2, matrix 256 × 192, NEX 3, duration 13 min). Probabilistic fiber tracking was performed using a dedicated software (*Brainance MD, Advantis Medical Imaging, Eindhoven, NL*). EPI correction tool for distortion correction was applied before image analysis. The fractional anisotropy threshold was set at 0.15, minimum fiber length 0 mm, maximum fiber length 200 mm, angular threshold 27°, and step size 1 mm. The dentatorubrothalamic tract (DRTT) was obtained by manual definition of three regions of interest (ROIs) on axial images: the cerebellar dentate nucleus ipsilateral to the target, the ipsilateral red nucleus, and the supposed location of the ipsilateral Vim at the level of the thalamus on the AC-PC plane (Fig. 2).

**Figure 3:**
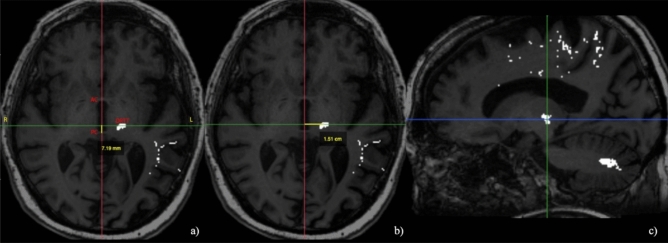
Direct AP (**a**), RL (**b**) and SI (**c**) coordinates manual measurement on multiplanar 2D reconstruction of the DRTT.

After DRTT reconstruction, direct coordinates were measured: (i) the mediolateral (RL) coordinate, defined as the distance from the center of the fiber tract to the AC-PC midline on the axial slice; (ii) the anteroposterior (AP) coordinate, defined as the distance from the PC line to the point where the RL coordinate intersected the AC-PC line on the axial slice; (iii) the superoinferior (SI) coordinate, defined as the distance from the center of the fiber tract to the plane passing through the AC-PC line on the coronal slice (Fig. 3).

All values were given in millimeters. Direct coordinates were measured by two neuroradiologists (with, respectively, five and 25 years of experience), blinded to the intraprocedural reports and the indirect coordinates values. In cases of discordance, a further consensus reading was performed.

### Statistical analysis

The measurements were recorded as mean values with standard deviations for all three spatial coordinates (AP, SI, RL). Statistical analysis was performed using MedCalc software (Ostend, Belgium, version 11.3 for Windows). The Bland–Altman test was performed to evaluate the agreement between direct and indirect coordinates compared to clinically effective coordinates. Limits of agreement between direct, indirect, and clinically effective coordinates were calculated, as well as confidence intervals of the differences between single coordinate measurements. The slope and intercept of the regression line that interpolated the variance of the targeting coordinate measurements in relation to the clinically effective coordinates were obtained. The median absolute percentage error was also calculated. The intraclass correlation coefficient (ICC) has been elaborated to evaluate the agreement between indirect and clinically effective coordinates and between direct and clinically effective coordinates (either on single or mean measurements).

All procedures performed in the study were in accordance with the ethical standards of the 1964 Helsinki Declaration and its later amendments, and approved by the Institutional IRB (University of L’Aquila, protocol number n. 01/2020). Informed consent was obtained from all individual participants included in the study. Identifying information about participants is not available in the article.

## Data Availability

The datasets generated during and/or analysed during the current study are available from the corresponding author on reasonable request.
